# Efficacy of Early Closed Toe Amputation for Toe Ulcers with Suspected Osteomyelitis after Revascularization for Chronic Limb-Threatening Ischemia

**DOI:** 10.3400/avd.oa.21-00136

**Published:** 2022-06-25

**Authors:** Tsunehiro Shintani, Sachi Suzuki, Naoya Kikuchi, Takumi Ariya, Kayoko Natsume, Kazuhiro Ookura, Jun Okui, Yasunori Sato, Hideaki Obara

**Affiliations:** 1 Department of Vascular Surgery, Shizuoka Red Cross Hospital, Shizuoka, Shizuoka, Japan; 2 Department of Plastic and Reconstructive Surgery, Shizuoka Red Cross Hospital, Shizuoka, Shizuoka, Japan; 3 Department of Cardiac Surgery, Shizuoka Red Cross Hospital, Shizuoka, Shizuoka, Japan; 4 Department of Preventive Medicine and Public Health, Keio University School of Medicine, Tokyo, Japan; 5 Department of Surgery, Keio University School of Medicine,Tokyo, Japan

**Keywords:** toe amputation, toe ulcer, chronic limb-threatening ischemia

## Abstract

**Objective:** In this study, we aim to evaluate the efficacy of early closed toe amputation on the wound management of toe ulcers with suspected osteomyelitis after revascularization for chronic limb-threatening ischemia (CLTI).

**Methods:** This retrospective study included patients who have underwent revascularization for toe ulcers associated with CLTI at Shizuoka Red Cross Hospital from 2015 to 2021. Wound management comprised early closed toe amputation for toe ulcers with suspected osteomyelitis (19 toes in 17 patients) or conservative treatment (35 toes in 26 patients). The primary endpoint was wound healing after revascularization. We compared the wound healing rate at 90 days and median healing time of early closed toe amputation versus conservative treatment.

**Results:** Compared with the conservative treatment, early closed toe amputation was able to achieve a better wound healing rate at 90 days (89.5% vs. 68.6%; P<0.01) and a shorter median healing time (19 days vs. 62 days; P=0.01).

**Conclusion:** There remains no established wound management for toe lesions associated with CLTI. Despite its several disadvantages including wound infection and possible foot deformity, early closed toe amputation for toe ulcers with suspected osteomyelitis can be considered a safe approach in terms of wound management.

## Introduction

As per the Global Vascular Guidelines, the restoration of straight-line blood flow to the foot is the primary goal of revascularization for chronic limb-threatening ischemia (CLTI) with tissue loss.^1)^ However, there remain no guidelines with regard to wound management after revascularization for CLTI. Potential foot complications include foot ulcer, cellulitis, abscess, foot gangrene, and necrotizing fasciitis. Despite toe ulcer being the mildest of these foot complications, there is yet no established optimal strategy to treat this condition. Although there are guidelines on the diagnosis and treatment of diabetic foot disease, some differences can be noted in terms of wound management of toe ulcers in patients with CLTI compared with diabetic foot.^2)^ First, the durability of increased blood flow after revascularization in CLTI has been deemed questionable. Previous Japanese studies have reported a high restenosis rate after endovascular revascularization for below-knee (BK) lesions, which are noted in most patients with CLTI.^3–5)^ Second, the diagnosis of osteomyelitis in the presence of ischemia is deemed uncertain. Osteomyelitis is suggested by the presence of exposed bone or a positive probe-to-bone (PTB) test result, but the test is known to have low sensitivity.^6)^ Magnetic resonance imaging has been identified as a reliable modality in the diagnosis of osteomyelitis in diabetic foot, but its efficacy in the presence of ischemia remains unclear.^7)^ Toe ulcer is sometimes associated with osteomyelitis, and wound healing is delayed under conservative treatment in these cases.^8)^ Third, there is some evidence that primary closure of toe amputation is a safe technique for diabetic foot.^9, 10)^ However, there is a paucity of evidence of primary closure of toe amputation for foot lesions associated with CLTI.^11, 12)^ Fourth, higher levels of amputation such as transmetatarsal amputation (TMA) or BK amputation may achieve better healing rates than toe amputation for foot lesions associated with CLTI, but these could also result in loss of walking ability. In a previous study, it was found that the ability to walk was maintained by 98% of patients with CLTI who underwent toe amputation compared with 86% of those who underwent TMA and 33% of those who underwent BK amputation.^13)^

Therefore, we hypothesized that early closed toe amputation for toe ulcers with suspected osteomyelitis after revascularization for CLTI can be useful in terms of wound management. Thus, in this present study, we evaluated the wound healing after revascularization between toe ulcers treated with early closed toe amputation versus conservative treatment.

## Materials and Methods

### Patients and study setting

This retrospective study included patients who have underwent revascularization for toe ulcers associated with CLTI at Shizuoka Red Cross Hospital from April 2015 to April 2021. Toes with ulcers and infections that exceeded the metatarsophalangeal joint were excluded. Patients who had less than 90 days of follow-up after revascularization were also excluded. CLTI was diagnosed when a toe ulcer was present for at least 2 weeks and was accompanied by a Wound, Ischemia, and Foot Infection (WIfI) system ischemia grade of ≥1 (Ankle Brachial Index <0.8 or skin perfusion pressure (SPP)<65 mmHg).^1)^

This study was approved by our institutional review board (with approval number 2019-19). Written informed consent was obtained from all patients.

### Revascularization procedure and wound management

Revascularization comprised endovascular treatment (EVT), bypass surgery, or hybrid surgery. Although the restoration of straight-line blood flow to the foot has been identified as the primary goal for CLTI with tissue loss, the correction of inflow (aortoiliac or femoropopliteal) disease alone may suffice to heal the toe ulcer in some cases. Revascularization procedure was selected based on the preference of one vascular surgeon (TS). Revascularization was performed by two board-certified vascular surgeons (TS and KN).

**Figure 1:**
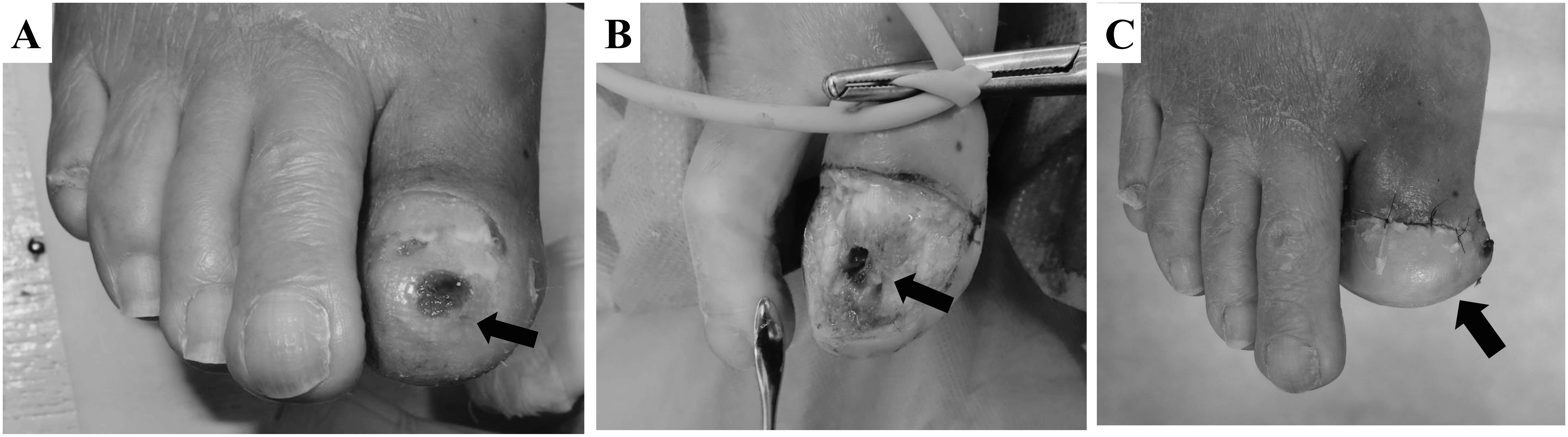
Fig. 1 Closed toe amputation for a toe ulcer with osteomyelitis. (**A**) The black arrow shows a toe ulcer on the right great toe. The probe-to-bone test result was positive. (**B**) Operative findings of toe amputation. The black arrow shows a pinhole at the distal phalanx that suggests osteomyelitis. (**C**) The black arrow shows the stump of the right great toe after resection of the distal phalanx.

Toe ulcers were treated by either closed toe amputation or conservative treatment after revascularization. Our wound management was as follows: When the PTB test came back positive, we clinically suspected osteomyelitis and thereafter performed closed toe amputation (**Figs. 1A** and **1B**). However, even if the PTB test was positive, conservative treatment was still selected in some cases where the toe ulcers were free from infection. The presence of toe infection was diagnosed by WIfI system foot infection grade of ≥1. Toe amputations were performed as soon as possible after revascularization. Early closed toe amputation was defined as toe amputation performed within 1 month after revascularization.

Our surgical technique for closed toe amputation comprised of soft tissue debridement (including the ulcer), resection of at least the distal phalanx, and primary closure of the wound. When infection extended over the distal phalanx or further osteomyelitis was suspected, we opted to resect the middle phalanx or the proximal phalanx. Toe amputations were performed either under local anesthesia or under general anesthesia when performed concomitantly with bypass surgery. After soft tissue debridement and bone resection, fish-mouth flaps were created, and the stump was loosely closed with interrupted sutures (**Fig. 1C**). A Penrose drain was indwelled until the next day. Sutures were removed at 2 weeks after toe amputation. Bone specimens resected during surgery were then sent for microbiological examination. When wound infection after closed toe amputation was suspected, the sutures were removed, and the wound was left open for further debridement or higher amputation.

Meanwhile, for conservative treatment, soft tissue debridement, or topical ointment application was performed. However, when it seemed that conservative treatment was not achieving wound healing, the treatment was converted to closed toe amputation. Closed toe amputations performed more than 1 month after revascularization were classified as conservative treatment in this present study.

The judgment of the PTB test and wound management were performed by one board-certified plastic and reconstructive surgeon (SS).

The study cohort did not include any patients with systemic infection or patients who received long-term (≥14 days) antibiotic administration within 1 month prior to revascularization. No specific antibiotic protocol was followed for either treatment method, except for antibiotic administration during the perioperative period after revascularization (1–3 days).

### Data collection and outcomes

Data were collected on patients’ demographics, comorbidities, wound status, ambulatory status, result of vascularization, duration of antibiotic therapy from revascularization to wound healing, and timing of toe amputation after revascularization. Wound status data included the involved toe, PTB test result, presence of toe infection, WIfI status, and preoperative SPP at the dorsal or plantar side of the foot. Ambulatory status was classified as either ambulatory or wheelchair. There were no bed-ridden patients reported in this present study. The result of revascularization included the achievement of one straight line of perfusion to the foot, achievement of a direct angiosome, and postoperative SPP. Furthermore, the proportions of BK lesions and EVT for BK lesions in patients with one straight line of perfusion to the foot were recorded. Hemodynamic success after revascularization was defined as a postoperative SPP of ≥40 mmHg on both the dorsal and plantar sides.^14, 15)^

The primary outcome was wound healing after revascularization. Wound healing was defined as the achievement of complete epithelialization on the affected toe. We then compared the wound healing rate at 90 days and the median wound healing time between the early closed toe amputation group and the conservative treatment group. Delayed wound healing was defined as healing that occurred more than 90 days after revascularization.

The secondary outcomes were wound complications (the need for further toe amputation in the early closed toe amputation group or the conversion to closed toe amputation in the conservative treatment group), ulcer recurrence after wound healing, and loss of ambulation after toe amputation in ambulatory patients.

### Statistical analysis

Unless otherwise indicated, continuous variables are presented as mean±standard deviation and were compared with t-test, whereas wound healing time was compared using Mann–Whitney U test. Categorical variables are presented as frequencies and percentages and were compared using Fisher’s exact test.

The difference in terms of wound healing rate between the early closed toe amputation group and conservative treatment group was assessed using Kaplan–Meier method with log-rank test.

All P-values were two-sided, and P<0.05 was taken to indicate a statistically significant difference. All analyses were performed using IBM SPSS version 24 (IBM Corp., Armonk, NY, USA).

## Results

### Patient population

**Figure 2:**
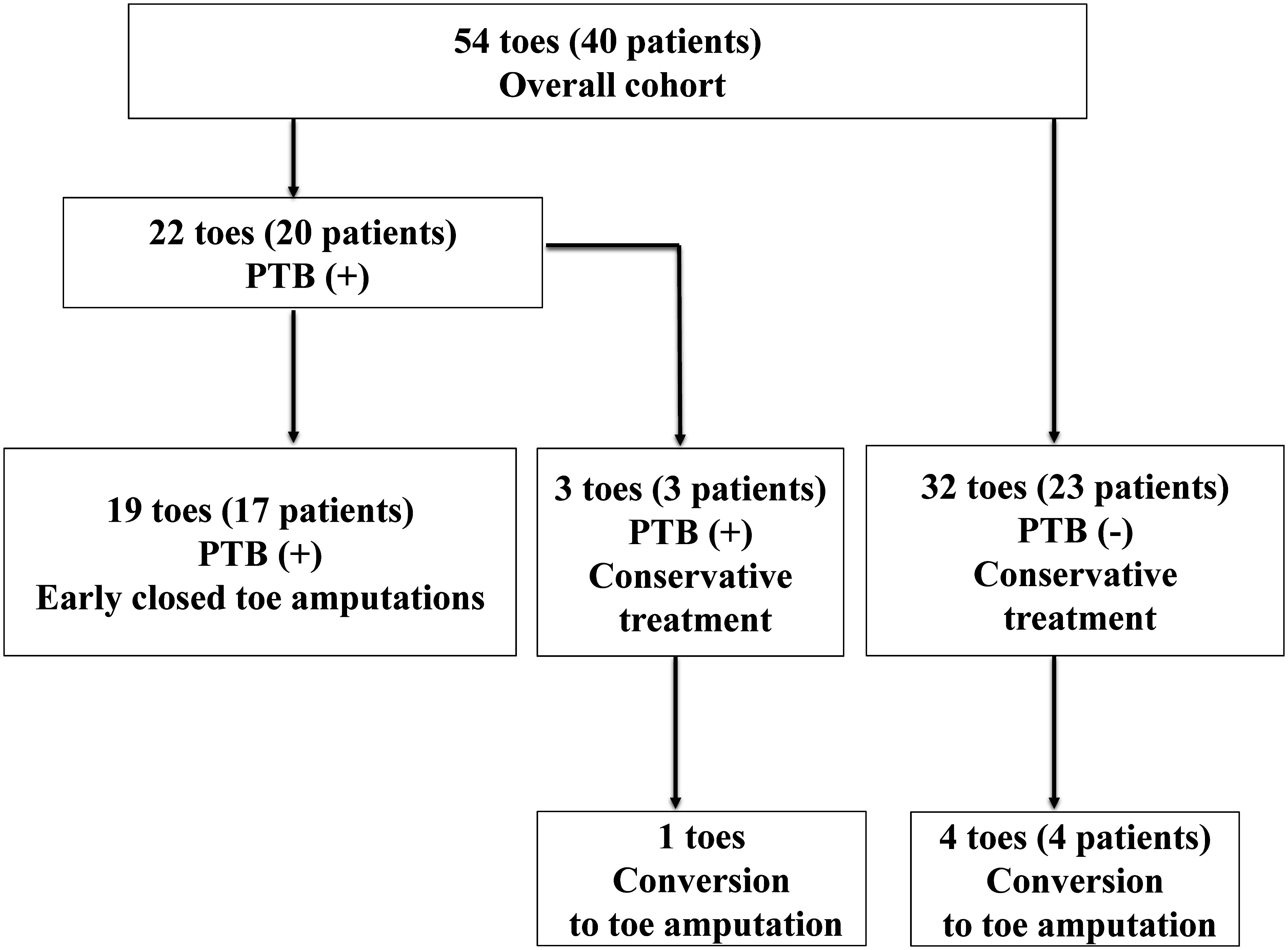
Fig. 2 Flowchart of patient inclusion and exclusion.

In total, 43 patients have underwent revascularization for toe ulcers associated with CLTI from 2015 to 2021. We excluded two patients who died and one patient who was lost to follow-up within 3 months after revascularization. The remaining 40 patients (54 toes) were deemed eligible for study inclusion. As per our findings, 22 toes (20 patients) were clinically suspected to have osteomyelitis (based on a positive PTB test). However, three toes underwent conservative treatment, as there was no toe infection present. The cohort was finally divided into the early closed toe amputation group (19 toes in 17 patients) and the conservative treatment group (35 toes in 26 patients) (**Fig. 2**).

PTB: probe-to-bone test

The demographics, comorbidities, wound status, ambulatory status, result of revascularization, and duration of antibiotic therapy in each group are summarized in **Table 1**. The early closed toe amputation group included more patients with toe infection than the conservative treatment group.

**Table 1:** Table 1 Baseline demographic data, comorbidities, wound status, result of revascularization, duration of antibiotic therapy, and outcomes

	Overall cohort (54 toes in 40 patients)	Early closed toe amputations (19 toes in 17 patients)	Conservative treatment (35 toes in 26 patients)	P-value
Demographic data				
Age, years	78.0±9.3	79.7±7.6	77.1±9.9	0.37
Male sex	30 (75.0)	12 (70.6)	20 (76.9)	0.73
Follow-up, months	23.8±20.9	21.8±19.2	24.5±21.7	0.68
Comorbidities				
Hypertension	26 (65.0)	8 (47.1)	19 (73.1)	0.11
Diabetes mellitus	20 (50.0)	6 (35.3)	15 (57.7)	0.22
Coronary artery disease	13 (32.5)	5 (29.4)	9 (34.6)	1.00
Cerebrovascular disease	10 (25.0)	5 (29.4)	5 (19.2)	0.48
Hemodialysis	17 (42.5)	7 (41.2)	12 (46.2)	1.00
Smoking history	30 (75.0)	13 (76.5)	19 (73.1)	1.00
Albumin, mg/dl	3.6±0.5	3.7±0.4	3.6±0.6	0.45
EF, %	65.7±11.0	67.8±7.9	63.7±12.5	0.23
Wound status				
Involved toe (I/II/III/IV/V)	22/12/3/6/11	8/3/2/1/5	14/9/1/5/6	0.55
WIfI status (I/II/III/IV)	3/5/15/17	2/2/5/8	1/3/12/10	0.64
PTB test positive	22 (40.7)	19 (100.0)	3 (8.6)	< 0.01
Toe infection	11 (20.4)	9 (47.4)	2 (5.7)	< 0.01
Preoperative SPP (dorsal)	27.3±16.4 (39 patients)	33.0±16.3 (17 patients)	23.0±15.3 (24 patients)	0.05
Preoperative SPP (plantar)	28.9±17.0 (39 patients)	31.7±15.8 (17 patients)	26.0±17.4 (24 patients)	0.30
Ambulatory status				
Ambulatory	33 (82.5)	12 (70.6)	23 (88.5)	0.23
Result of revascularization				
One straight line	32 (80.0)	15 (88.2)	19 (73.1)	0.28
BK lesion	23 (71.9) (32 patients)	11 (73.3) (15 patients)	13 (68.4) (19 patients)	1.00
BK EVT	13 (56.5) (23 BK lesions)	6 (54.5) (11 BK lesions)	7 (53.8) (13 BK lesions)	1.00
Direct angiosome	24 (61.0)	10 (58.8)	16 (61.5)	1.00
Postoperative SPP (dorsal)	50.1±21.5 (37 patients)	51.6±15.8 (15 patients)	49.0±24.4 (24 patients)	0.71
Postoperative SPP (plantar)	46.4±19.9 (36 patients)	45.7±14.7 (15 patients)	46.0±22.5 (23 patients)	0.96
Hemodynamic success	20 (54.1) (37 patients)	9 (60.0) (15 patients)	12 (50.0) (24 patients)	0.74
Duration of antibiotic therapy (days)	6.70±7.7	8.59±9.42	4.58±5.51	0.13
Outcomes				
Achievement of wound healing	52 toes in 38 patients (96.3)	19 toes in 17 patients (100.0)	33 toes in 24 patients (94.2)	0.54
Wound healing time (days)	48.0 [13.6] (19.0–88.0)	19.0 [1.4] (15.0–25.0)	62.0 [8.3] (39.0–111.0)	0.01
Delayed wound healing	12 (22.2)	1 (5.3)	11(31.4)	0.04
Further toe amputation	NA	2(10.5)	NA	NA
Conversion to toe amputation	NA	NA	5 (14.3)	NA
Timing of toe amputation (days)	NA	0.0 [1.1] (0.0–6.0)	148.0 [14.1] (136.0–179.5) (5 toes)	NA
Ulcer recurrence	9 of 38 patients (23.7)	6 of 17 patients (35.3)	4 of 24 patients (16.7)	0.27

Data are presented as mean±standard deviation for continuous variables and as n (%) for categorical variables. SPP data are missing for some patients. Wound healing time and timing of toe amputation are presented as median [standard error] (interquartile range). Involved toe, PTB test positivity, toe infection, and outcomes except for ulcer recurrence are toe-based data. EF: ejection fraction; PTB: probe-to-bone; SPP: skin perfusion pressure; BK: below-knee; EVT: endovascular treatment; NA: not applicable

### Outcomes

**Figure 3:**
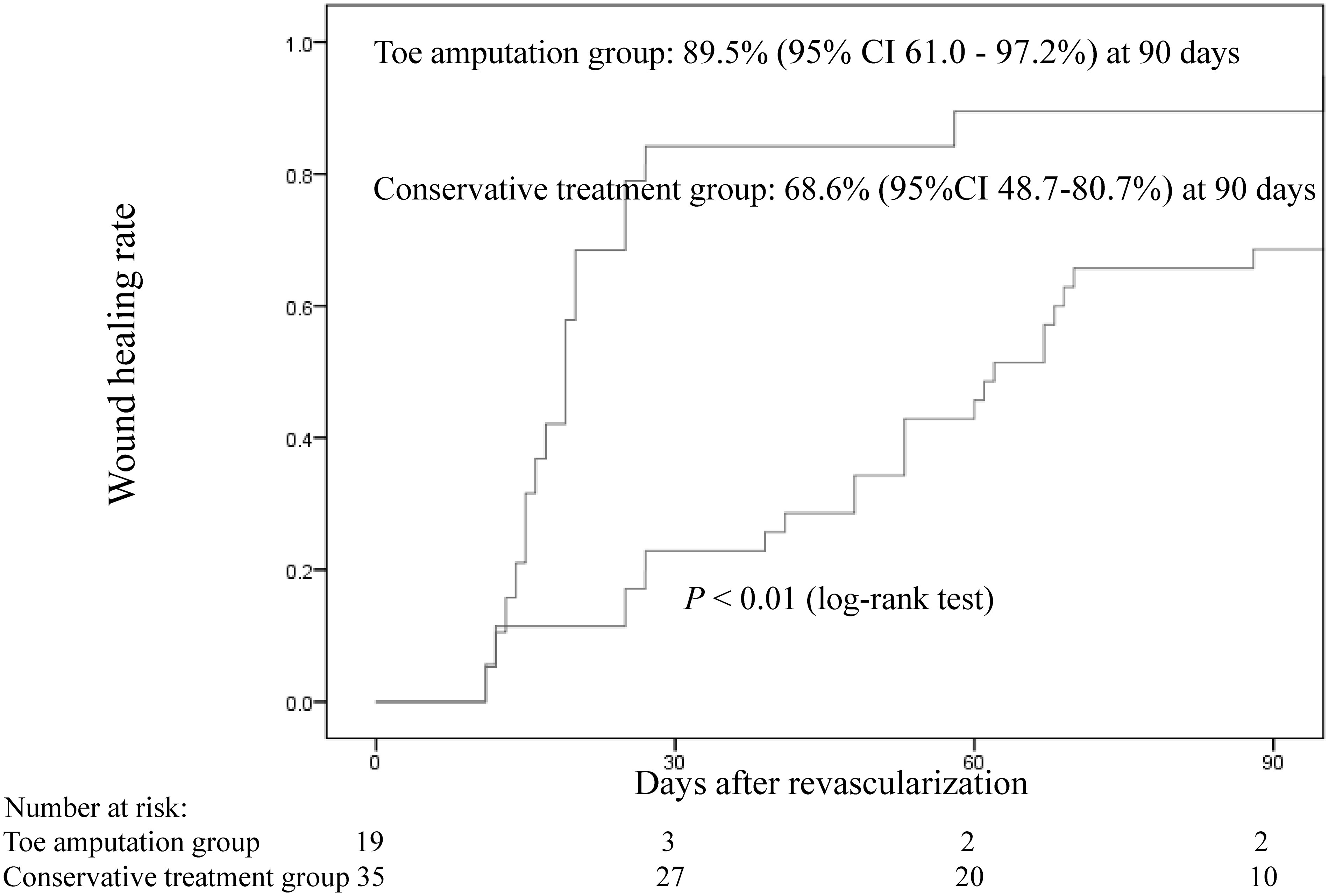
Fig. 3 Kaplan–Meier curves showing the wound healing rates of early closed toe amputation versus conservative treatment for toe ulcers after revascularization for CLTI.

As per the results of this study, the healing rate at 90 days was found to be better in the early closed toe amputation group than the conservative treatment group (89.5% (95%CI 61.0–97.2%) vs. 68.6% (95%CI 48.7–80.7%); P<0.01) (**Fig. 3**). The outcomes of early closed toe amputation versus conservative treatment are shown in **Table 1**. Wound healing was achieved in all toes except for two toes in the conservative treatment group. The overall median wound healing time of toe ulcers was 48 days. The median wound healing time was significantly shorter in the early closed toe amputation group than the conservative treatment group (19 days vs. 62 days; P=0.01). More cases of delayed wound healing were noted in the conservative treatment group than the early closed toe amputation group. The details of the cases with delayed wound healing are shown in **Table 2**. Conversion to toe amputation was required for five toes in the conservative treatment group; all of these cases (Cases 3, 5, 9, 10, and 11) were able to achieve wound healing within 90 days after toe amputation (13, 57, 12, 19, and 20 days). Case 5 required repeat EVT for re-occlusion of a BK lesion. The patient in Case 9 was suspected to have osteomyelitis, but received conservative treatment. In the early closed toe amputation group, two patients required further toe amputations due to wound infection. The wound healing was delayed by more than 90 days in one case (Case 12). The median timing of toe amputation in the early closed toe amputation group was 0 days after revascularization. We then performed concurrent toe resection with revascularization in 11 of 19 toes. There were nine cases of ulcer recurrence after wound healing. Among these recurrent ulcer cases, two were considered to be related to foot deformity after toe amputation. The resected bone specimens in the early closed toe amputation group all returned positive microbiological results. However, the resected bone specimen returned a negative microbiological result in one of the five cases (Case 11) in the conservative treatment group that underwent conversion to amputation. Moreover, none of the 16 ambulatory patients who underwent toe amputation experienced loss of ambulation after toe amputation.

CLTI: chronic limb-threatening ischemia

**Table 2:** Table 2 Details of cases of delayed wound healing of toe ulcers

Case	Toe	PTB	Details of treatment	Outcome
1	I	−	Conservative	Wound healing at POD 237
2	I	−	Conservative	Wound healing at POD 111
3	II	−	Conservative → toe amputation (distal phalanx) at POD 139	Wound healing at POD 152 (*13)
4	I	−	Conservative	Nonhealing at POD 180
5	V	−	Conservative → toe amputation (distal and middle phalanx) at POD 148	Wound healing at POD 205 (*57)
			→ re-EVT (BK lesion) → toe amputation (proximal phalanx) at POD 154	
6	II	−	Conservative	Wound healing at POD 109
7	V	−	Conservative → lost to follow-up	Nonhealing at POD 111
8	V	+	Conservative	Wound healing at POD 97
9	II	+	Conservative → toe amputation (distal phalanx) at POD 211	Wound healing at POD 223 (*12)
10	IV	−	Conservative → toe amputation (distal and middle phalanx) at POD 148	Wound healing at POD 167 (*19)
11	IV	−	Conservative → toe amputation (distal and middle phalanx) at POD 133	Wound healing at POD 153 (*20)
12	V	+	Early closed toe amputation (distal, middle and proximal phalanx) at POD 0	Wound healing at POD 180
			→ soft tissue debridement for wound infection at POD 14	

* Days of conservative treatment from toe amputation to wound healing. PTB: probe-to-bone test; Conservative: conservative treatment; POD: postoperative day; EVT: endovascular treatment; BK: below-knee

## Discussion

Although many authors have reported the technique and results of revascularization, studies on wound management after revascularization for CLTI have remained to be extremely rare. Some authors advocate early primary closure or concurrent foot tissue resection (including debridement and minor amputations) after revascularization for CLTI.^11, 12)^ We have focused on the wound management of toe ulcers after revascularization for CLTI. Our study showed that better wound healing after revascularization was achieved by early closed toe amputation for suspected osteomyelitis compared with conservative treatment.

Early closed toe amputation for suspected osteomyelitis with CLTI is known to have several advantages. Primary closed toe amputation achieves wound coverage with an intact skin barrier (leading to quicker wound healing), and bone resection reduces the wound tension, which can be useful for toes with thin subcutaneous tissue.^9, 11)^ The first advantage of early closed toe amputation is that quicker wound healing is especially important for patients with CLTI with BK lesions, which are often treated by EVT. The reported incidence of angiographic stenosis at 90 days after infrapopliteal angioplasty in patients with CLTI is 73%.^3)^ About 70% of patients with CLTI in our study had BK lesions, and about 50% of these BK lesions were treated by EVT. In such a population, wound healing can get more delayed unless wound healing is achieved before the restenosis of the treated lesion. In our study, one patient in the conservative treatment group needed repeat EVT to achieve wound healing.

The second advantage is that toe amputation eliminates possible osteomyelitis and reduces the duration of antibiotic treatment. In this present study, toe amputation was aggressively performed for toe ulcers with clinically suspected osteomyelitis. Several studies have evaluated the management of diabetic foot osteomyelitis by medical (long-term antibiotic treatment) or surgical treatment (infected bone resection).^16–18)^ However, there remains lacking available evidence to identify the best treatment for diabetic foot osteomyelitis.^16)^ Only one randomized controlled trial has showed that the wound healing and minor amputation rates were the same after antibiotic treatment alone versus surgical treatment in patients with diabetic foot osteomyelitis^17)^; in this previous trial, the duration of antibiotic treatment was 90 days in the antibiotic treatment group, while only postoperative empirical antibiotics were administered in the surgical treatment group. In our study, the conservative treatment group did not receive long-term antibiotic therapy. Of the five cases of conversion to toe amputation in the conservative treatment group, four were suspected to be persistent osteomyelitis according to the microbiological result. These cases of persistent osteomyelitis may have been eradicated by long-term antibiotic treatment. However, as wound healing was achieved within 90 days in all cases after conversion to toe amputation, early toe amputation might have been useful in these conversion cases.

There are also several potential disadvantages of early closed toe amputation for patients with CLTI.^18)^ First, closed toe amputation may increase the risk of wound infection, which then leads to a higher level of amputation. A previous study on toe amputation in the diabetic foot recommended the secondary closure of toe amputation when infection control is not achieved.^10)^ In all cases in the early closed toe amputation group in this present study, we have determined that infection control was achieved based on the operative findings and therefore chose to perform primary closure. However, two of these patients required further toe amputations due to wound infection. Wound healing was finally achieved in these two patients.

The second disadvantage of early closed toe amputation is the fear of postoperative foot deformity and the development of an ulcer in association with this deformity.^17–19)^ A previous study stated that toe amputation is associated with minimal functional disability.^10)^ In our study, there were two cases in which the ulcers were considered to be associated with foot deformity after toe amputation; wound healing was achieved under conservative treatment in these two cases. Another possible disadvantage of toe amputation is a potential decline in walking ability. However, in our study, no patients developed loss of ambulation associated with toe amputation. Further study is needed to evaluate the long-term effect of toe amputation on foot deformity, ulcer development, and ambulation.

It remains difficult to diagnose osteomyelitis in the presence of ischemia. Even after revascularization, magnetic resonance imaging has a very low sensitivity to diagnose osteomyelitis.^7)^ Thus, it is difficult to identify the presence of osteomyelitis before toe amputation for a toe ulcer associated with CLTI. In this present study, we identified the presence of osteomyelitis based only on the PTB test result. Therefore, the third disadvantage of early closed toe amputation for suspected osteomyelitis is the possibility of unnecessary bone resection. In our study, the microbiological culture results were positive for all resected bone specimens in the early closed toe amputation group, but these may have been false positives because of contamination by wound-colonizing flora.^20)^ Because we did not perform histological examination of the resected specimens, it is difficult to confirm the final diagnosis of osteomyelitis in our cases. There is a need for a more reliable imaging modality with which to detect osteomyelitis in the presence of ischemia.

Our study has focused on the wound healing of toe ulcers that were classified as Rutherford 5. In previous studies, the reported median wound healing times of Rutherford 5 wounds are 47 days (for nonhemodialysis patients after bypass surgery), 73 days (for hemodialysis patients after bypass surgery), and 96 days (for infrapopliteal lesions after EVT).^21, 22)^ Although the patients in these previous studies had different background characteristics to our patients, the overall median healing time in our study was 48 days, which is deemed comparable to the reported healing times.

This study has several limitations. First, it was a retrospective, single-center study. We did not evaluate the wound healing in patients who received conservative treatment for suspected osteomyelitis; thus, this warrants investigation in multicenter studies including large numbers of patients who receive conservative treatment for toe ulcers with suspected osteomyelitis. Second, the judgment of the PTB test was made by one surgeon, resulting in selection bias. Third, we had no established strategy regarding the timing of toe amputation after revascularization and the duration of antibiotic therapy. Our study was exploratory, and further studies under a strict protocol are necessary to determine the role of early closed toe amputation for toe lesions with suspected osteomyelitis associated with CLTI.

## Conclusion

There is yet no established wound management strategy for toe lesions associated with CLTI. In this study, we demonstrated that better wound healing after revascularization was achieved by early closed toe amputation for suspected osteomyelitis compared with conservative treatment. Although toe amputation has several disadvantages (including wound infection and possible foot deformity), early closed toe amputation for suspected osteomyelitis using the PTB test may be a safe approach in terms of wound management for toe lesions associated with CLTI. Further studies are necessary to confirm this result.
